# I Buy Medicines From the Streets Because I Am Poor: A Qualitative Account on why the Informal Market for Medicines Thrive in Ivory Coast

**DOI:** 10.1177/00469580221086585

**Published:** 2022-03-21

**Authors:** Armel Dagrou, Victor Chimhutu

**Affiliations:** 1 87364 University of Bergen, Department of Health Promotion and Development, Bergen, Norway; 2 Inland Norway University of Applied Sciences, Department of Public Health and Sports Sciences, Elverum, Norway

**Keywords:** pharmaceutical drugs, sub-Saharan Africa, ivory coast, cote d'Ivoire, informal providers, qualitative case study, informal markets, informal medicines

## Abstract

The informal market for medicines has been growing. In Ivory Coast, this informal market is an unofficial core part of the health system. Given the risks associated with the informal market for medicines, it is important to understand why this market continues to grow. It becomes even more important in the context of COVID-19, as a huge chunk of falsified medical products end up at the informal market. A qualitative case study design was chosen for this study, with in-depth interviews (IDIs) and focus group discussions (FGDs) being the methods for data collection. 20 IDIs and 3 FGDs were conducted. Participants in this study are sellers, buyers, and pharmaceutical experts. We found out that the informal market for medicines thrives because it is highly accessible, convenient, affordable, and that it is used for various social, cultural, and religious reasons. The study concludes that although this informal market presents a clear danger to public health, it is thriving. For authorities to address this public health challenge, there is need for a holistic and multi-pronged approach, which includes addressing health systems factors and strengthening regulatory framework.

**What do We Already Know About This Topic?**
We already know that informal providers in health care plays an important role in sub-Saharan Africa and that the informal market for medicines is growing in the region.
**How Does Your Research Contribute to This Field?**
The research looks in-depth into the factors and reasons why this market continues to be attractive to users despite the public health threat it poses considering that most substandard and falsified pharmaceutical products end up on these informal markets for medicines.
**What Are Your Research’s Implications Towards Theory, Practice, and Policy?**
The research is of significance because it provides evidence that the informal market is thriving and that the level of user acceptability is high. In this regard, the study shows that there is a ready market for substandard and falsified pharmaceutical products, something that presents a clear public health danger to the vulnerable people using these markets.

## Introduction

In many low-income countries (LICs), the informal sector plays a big role in the provision of health care.^1^ This article focuses on the informal market for medicines, one example of informal health providers.^2^ We will use the case of one popular informal market for medicines in Ivory Coast, *Roxy*, to investigate why this informal market continues to grow in popularity as is the trend in many LICs.^1-3^

Access to health care and medicines is a human right and an obligation of the states.^4^ The right to health becomes even more central as the central principle of the *2030 Agenda for Sustainable Development* is to ensure that no one is left behind.^5^ However, many LICs still fail to provide adequate medicines to their populations.^1^ This failure by states in their obligation encourage people, especially the poor, to use the informal market.^1,6^ Although lack of adequate provision by the state and formal actors is among the main reasons for the steady growth of the informal market for medicines, there are a number of other reasons including it being economically attractive to users,^1,7,8^ and also that this market is socially and culturally acceptable in these contexts.^2,8^ The informal market for medicines is also seen as complementary to the formal system, especially in providing care to populations in remote and inaccessible areas.^9^ In this regard, informal market for medicines, unofficially is core in the health systems of many developing countries.^6,7,10^ The sudden expansion of this market is mainly driven by demand and weak legal and regulatory frameworks.^3,11,12^

This study becomes imperative especially in the context where this exponential growth of the informal market for medicines is also associated with an increase in falsified medical products which provide a great risk to the population.^3,11,13^ According to World Health Organization, 1 in 10 medical products in low-and middle-income countries are substandard or falsified^1^ and most of these products are on the informal market.^3,11^ In the case of malaria only, it is estimated that substandard and falsified anti-malarial drugs could be causing over 115 000 more deaths in sub-Saharan Africa.^1^ This is also compounded by the fact that trading in falsified medicines is an illicit “profitable” business racking over US$200bn annually,^3^ with the Africa region being the most targeted market, where up to 42% of all falsified medicines are sold.^1^

These high figures are unsettling especially in the context of COVID-19 and where reports of falsified vaccines are surfacing from the Africa region.^14,15^ Although falsified or substandard medicines also find their way into the formal system, for example, falsified chloroquine, a much-touted possible COVID-19 remedy that was seized in Cameroon,^11^ it is undeniable that most of these products end up on the informal market.^1,3^ It therefore, becomes significant to investigate why people prefer the informal market over the formal market, this understanding goes a long way in helping policy makers to coming up with appropriate courses of actions and regulatory frameworks to this pressing public health challenge.

### Theoretical Framework

To better understand the factors leading people to buy and participate in the informal market for medicines, we will use the AAAQ model (Availability, Accessibility, Acceptability, and Quality). The framework comes from a rights perspective^16^ and has been used in health-related research that deals with provision, access, and utilization of health services and products.^17^ The use of this framework in this study, is informed by the fact that access to medicines is a rights issue. In order to achieve global development goals linked to health, equitable access to medicines must be high on the agenda at local, national, and international levels. Therefore, using a framework like AAAQ from a rights perspective on this topic of informal markets for medicines, elevate the importance of the matter. Access to medicines is not a matter of choice but must be seen as a fundamental human right, equally when poor people resort to access medicines from the informal market, it must be investigated befittingly from a rights perspective. The first A in the framework represents availability, meaning services, and medical products must be available in adequate numbers. The second A represents accessibility, meaning services, and medical products must be accessible in terms of affordability, geography, and without any discrimination. The third A represents acceptability, meaning medical services and products must be acceptable socially and culturally. The Q stands for quality, meaning medical services and products must of good quality and safe to use. Quality in the context of this study is defined by the perceptions and experiences of pharmaceutical experts who participated in this study. Likewise, users and sellers of medicines at the informal markets also had their perceptions on quality. We will present these perceptions with thick descriptions to facilitate for the readers to make informed choices on whether these interpretations suit their own understanding or contexts.

Although the AAAQ framework is useful in explaining these four dimensions on access to medicines it must be noted that it has serious limitations in cases where these informal markets are used for illicit recreational drug. Informal markets for medicines, such as *Roxy* are also known as hubs for recreational drugs that cause addiction. Our use of the AAAQ is only in health promoting ways, where vulnerable populations use informal markets in genuine attempts to improve their health statuses.

## Methods

### Study Setting

*Roxy* market is located in Adjamé, Abidjan. Abidjan is one of the major cities in West Africa and the economic capital of Ivory Coast. This port city is known for having one of the biggest informal markets for medicines in West Africa, which is in Adjamé.^11^ Adjamé is the main commercial hub in Abidjan with its biggest market called *Adjamé* market. The largest bus terminus in Ivory Coast is located in Adjamé and being also in a coastal city, Adjamé connects to many destinations within Ivory Coast and in the region. This makes goods including medicines sold on the informal market easily accessible. *Roxy* market is exclusively dedicated to the selling of medicines. Although they are other areas near Adjamé where medicines are sold on the streets, *Roxy* market is by far the biggest and busiest informal market for medicines in Ivory Coast and arguably in the region. *Roxy* is also one of the main supply points for other informal markets in the country and region.

It is against this background that we purposefully selected *Roxy* market in our attempt to investigate and understand the reasons why people buy medicines from the informal market. This purposeful selection of the study site also made it relatively easy for us to have access to the users of and sellers at this market. Additionally, *Roxy* gave us the opportunity to observe and have a deeper understanding of the activities and interactions at a market of this nature in its natural setting, which at latent level could have influenced our own interpretations in this study.

### Study Design

A qualitative case study design was chosen for this study. The study was focusing on one of the popular informal markets for medicines in Ivory Coast, *Roxy* presented and described above. The study targeted three groups of participants, the sellers, the buyers, and pharmaceutical experts. The experts were selected based on having knowledge on the pharmaceutical industry, the informal market, and regulatory frameworks. Of the six pharmaceutical experts in this study, three were trained as pharmacists, two as pharmacy technicians and one as a medical doctor. In terms of gender, three of the experts were females and the other three males and their age was between 35 and 55 years. The first two pharmaceutical experts were recruited through the Directorate of Pharmacies, Drugs and Laboratories (DPDL) and thereafter snowballing techniques was used. Selection of the research participants was purposive. The purposive selection of the study site and research participants enabled us to have access to information that answers the study aim, which we may not have gotten had we made other choices.^18^

These three groups of participants, the sellers, buyers, and pharmaceutical experts, provided rich information to the study. This triangulation of the study participants also ensures and improves the quality of the study.^19^ As this was somewhat a sensitive topic, as the informal market for medicines in Ivory Coast is largely unregulated and therefore illegal, recruiting the sellers and buyers was challenging in the beginning, however, the study then used snowballing technique. Building rapport with research participants was also of importance in this study to establish trust, especially in the case of the sellers. In this study, we managed to build this rapport also because one of the authors has an in-depth understanding of the context and this insider perspective helped in the establishment of trust.

### Data Collection

Data collection for the study was done in two phases, in January of 2019 and from December 2019 to January 2020. Data that were collected for the first phase was mainly used in a master thesis of the main author,^20^ however, after realizing the richness of the data and its importance in the field, we decided to collect additional data. In-depth interviews (IDIs) and focus group discussions (FGDs) were used as the main methods for data collection. These two methods were used because of their conversational nature, we needed to understand the reasons why people use the informal market to buy medicines, and, in that regard, the interview method was more suitable. FGDs were also used with buyers because in a somewhat sensitive topic, users of the service may feel directly targeted in an interview and therefore reluctant to share their experiences, whereas in FGD, they can give generalized experiences of the group and thus less personal.^21^

In total, 20 IDIs were conducted, six with pharmaceutical experts and fourteen with the informal medicine sellers. Eleven of these were conducted during the second phase of data collected, that is, between December of 2019 and January of 2020, while nine were conducted during the first phase. Table 1 gives an overview of IDIs with pharmaceutical experts and sellers.

**Table 1.** Overview of IDIs with Pharmaceutical Experts and Sellers.

Category of Informants	Interviews 1st Phase	Interviews 2nd Phase	Total
Sellers	7	7	14
Pharmaceutical experts	2	4	6
**TOTAL**	**9**	**11**	**20**

A total of three FGDs were conducted with the buyers, and two of these were done during the first phase of data collection and one during the second phase. The three FGDs had thirteen participants. Of these thirteen participants, four were women and nine were men, participants were not separated by gender during the FGDs as the topic was not gender sensitive. Table 2 gives an overview of FGDs.

**Table 2.** Overview of FGDs with buyers.

Name	Age	Sex	Occupation	FGD
Abdul	34	Male	Unemployed	FGD 1
Paul	31	Male	Taxi driver	FGD 1
Sadiki	33	Male	Sim card seller	FGD 1
Serge	29	Male	Mechanic	FGD 1
Edmond	41	Male	Security guard	FGD 1
Solange	25	Female	Hair dresser	FGD 2
Aminata	40	Female	Vendor	FGD 2
Joel	38	Male	Sidewal couturier	FGD 2
Stephane	27	Male	Unemployed	FGD 2
Theresa	35	Female	Vendor	FGD 3
Gisele	31	Female	Shop assistant	FGD 3
Ange	43	Male	Taxi driver	FGD 3
Lissana	28	Male	Unemployed	FGD 3

All IDIs and FGDs were conducted in French, the official language of Ivory Coast. The interview and topic guides covered the following thematic areas: experiences and perceptions of the informal drug market, the reasons, risks, and benefits attached to buying or selling at the informal market for medicines.

### Analysis

All IDIs and FGDs were voice-recorded, after permission was sought for and granted by the participants. After the data collection process, data were transcribed in French and then translated to English. After this stage the analysis of data began in NVivo 12, using thematic analysis.^22^ The transcripts were subjected to a thorough review by both authors before the coding exercise began. The first few interviews were coded separately and then we met and discussed the coding framework. This was an attempt to establish inter-coder reliability. Themes that emerged during this process constitutes the results of this study.

### Research Ethics

Ethical clearance was sought and granted in Norway by the Norwegian Centre for Research Data (NSD) and in Ivory Coast permission was granted to conduct the study by the Directorate for Pharmacies, Drugs and Laboratories (DPDL). Participation was voluntary. Most of the participants gave written consent while in few cases consent was given orally. Informants were assured of the highest possible levels of confidentiality and anonymity. We have depersonalized data by giving research participants pseudo-names.

## Results

In this section we will present the study findings. The main reasons that emerged for using the informal market for medicines are that: it is cheaper, the pricing is flexible, it is used in favor of the formal market which is heavily regulated, it is used for cultural, social, and religious reasons and finally the market thrives because it is a source of employment.

### Cheaper and Convenient

Throughout the FGDs, the buyers overwhelmingly expressed their favor towards buying drugs from the informal market. One of the main reasons for this was that buyers saw this market as a cheaper alternative to the formal market.

*“It's undeniable, the drugs at Roxy are cheaper. For poor people like me, who do not have a stable source of income, we cannot afford to buy drugs at the pharmacy, it makes sense to buy drugs at Roxy.” (Stephane, Buyer- FGD 2)*

Joel another participant agreed with Stephane that medicines are cheaper at the informal market but also went further to state that the informal market is far more convenient to use than the formal market, with or without a health insurance:

*“My wife and I have a health insurance, I have glaucoma in my left eye so I have to buy drugs every month and this medication is very expensive. We had to pay 18 000 CFA francs each, every month for a year and the insurance was also covering the drugs. I used to buy drugs at the pharmacy with my money and wait about one month before the insurance pays me back 25% of the price. One day I found the drugs I use for my treatment at Roxy and from that day I stopped paying for the insurance.” (Joel, Buyer- FGD 2)*

Sidiki, another participant, also noted that it is this convenience of the informal market that makes it attractive and a cheaper option. He emphasized the fact that at the informal market one can buy the exact quantities they want for immediate use:

*“Last time, my aunt went to the hospital and the doctor prescribed her tablets to take in the morning and in the evening. She had to take those tablets for 4 days to get better. In pharmacies, a pack of 24 of these tablets is sold for more than 7000 Franc CFA (approx. US$12). I went to Roxy and bought 12 of these tablets at 4000 Franc CFA (approx. US$7). As you can see, at Roxy you can buy the actual units you want whereas in pharmacy you are forced to buy a full pack” (Sidiki, Buyer- FGD 1)*

The fact that the informal market allows for the selling of drugs is smaller quantities and units can be convenience and attractive for those with less income. However, the perception that drugs are cheaper on the informal market was contested by Raymond, a pharmaceutical expert:

*“The argument most often mentioned by the buyers is that Roxy is cheaper, this is not necessarily true. For example, on the black market, the tablet cac-1000 is sold retail at a price of 200 or 250 francs (approx. US$0.45) while in pharmacy, the box of 10 tablet costs around 1800 francs (approx. US$3). Thus, anyone who does not know this or cannot afford to buy the full box, will buy it at Roxy but when you consider the cumulate value you realize they spend more. Twice as expensive because the conservation of the drug on the black market is not optimal which causes a loss in terms of quality” (Raymond, Pharmaceutical Expert- IDI)*

Although Raymond’s argument on pricing can be sustained, it must be noted, however, that it is the convenience and flexibility which make the informal market easily accessible, for example, the fact that at Roxy customers can buy medication in units and not packages, make it seemingly cheaper for customers. This flexibility is not available in the formal market. Many buyers prefer the informal market for its convenience even if the issue of affordability can be contested. Even Raymond confirmed this:

*“Some people choose the easy way. They tell themselves that at the pharmacy there is a lot of hassle with the prescriptions, or that insurances do delay in reimbursements. In this regard when they get the drug at seemingly reasonable prices on the black market, they go for it.” (Raymond, Pharmaceutical Expert- IDI)*

### Pricing and Terms of Payment Are Flexible on the Informal Market

Solange, a participant in FGD reported that at Roxy, they can negotiate for pricing, and get some discounts or credit facilities:

*“Pharmacies are complicated and rigid. When I go to Roxy, I always go to the same stall, I have a favourite seller. I have been going there for years now. Since I always go there, we now know each other well and we have even become friends. I always go to her booth because sometimes she gives me discounts. Sometimes she even gives me drugs on credit, and I can pay later when I earn money. I am also a vendor like her, so we understand and help each other.” (Solange, Buyer- FGD 2)*

The formal drugstores do not offer this kind of service. Therefore the informal market has this substantial advantage.

### Strict Adherence to Procedures by the Formal Market

Although this could be considered a positive thing in many contexts, paradoxically, it emerged that the strict adherence to the protocol by the formal market was perceived by buyers as among the reason why they prefer the informal market. The informal market was associated with convenience and flexibility:

*“Pharmacies are too complicated, most of the time they will ask you for a prescription, for you to get that prescription you need to visit a doctor, then you have to pay for consultation fees, it makes the whole process long and expensive. Sometimes they will tell you that the drug you are looking for is out of stock. It is very exhausting, frustrating and time consuming for people most of the time.” (Paul, Buyer- FGD 1)*

Paul here is raising many issues related to the convenience of the informal market and costs associated with using the formal market. It also follows that due to many costs associated with formal channels, many users of the informal market indulge in self-diagnosis, something potentially fatal. Aminata, a participant in FGD 2, is one person who engages in self-diagnosis: *“not everyone can afford to see a doctor and get a prescription. As a result, we just have to use Roxy to save time and money”*

Although there is a perception among the users that it is cheaper and convenient to buy drugs at *Roxy*, Raymond, a pharmaceutical expert emphasized that using medical products from the informal market is costly to the health of consumers. He cited a sad case of death that was recent:

*“I do not know if you have heard about it but there is a lady who died while going to the field, this story made a lot of noise in the news but there is no judicial follow up to this case. To put it in a nutshell, this woman died on her way to work in the field and the postmortem revealed that she regularly consumes drugs that she was getting from the black market, but this drug is not supposed to be taken on a regular basis as it is dangerous” (Raymond, Pharmaceutical Expert- IDI)*

This case by Raymond highlighted the dangers of self-medication and diagnosis, which naturally explains why the formal pharmaceutical industry need to adhere strictly to existing regulatory frameworks. Besides death, Raymond also raised the issue that over-consumption of non-prescribed drugs is causing a public health crisis of addiction in Ivory Coast, especially among the youths:

*“Medications can also create dependencies. Tramadol is killing young people in Ivory Coast. You will see, in a few years the government will have to create hundreds of rehabilitation centers for these young people who are taking these drugs for fun. The government has opened more than 100 dialysis centers to treat kidney failure. Specialists are saying these diseases are often due to intensive use of unapproved drug. The problem must be fixed at its source. Closing this black market would solve many problems including self-medication and the informal sale of psychotropic medication.” (Raymond, Pharmaceutical Expert- IDI)*

In this vein, it is clear that perceptions of users, sellers, and pharmaceutical experts fundamentally differ on whether strict procedures to medication is good or not. The pharmaceutical experts emphasize on the dangers on if it while users mainly emphasize that the informal market which is unregulated makes the process of acquiring medicines faster and cheaper.

### Cultural, Social, and Religious Reasons

It also emerged that there were some cultural, social, and religious reasons for using the informal market. Lassina stated that he prefers to go to the informal market because it also sells traditional herbs:

*“Another good thing about Roxy is that I can find traditional drugs and other stuff that allow me to perform better with my wife (some laughter from the group). This kind of stuff is not found in pharmacies. Anyways (laughing), I never tried to find them in pharmacies as Roxy is my place for that.” (Lassina, Buyer- FGD 3)*

From Lassina, we can see that traditional herbs are part of the reasons why he uses this market. Some of these traditional herbs, may be considered illegal on the formal market and yet they may have some significance in local settings. As such cultural beliefs and practices, such as boosting of virility plays a part in the utilization of the informal market. Users also raised an important issue that in cases where diseases attract social stigma, for example, sexually transmitted diseases (STIs), it was much safer for them to buy medication at the informal market discreetly:

*“Some people are so ashamed of their illness that they prefer to buy drugs at Roxy on the sly (discreetly). Sometimes, even the spouse may not be aware of this disease. This makes Roxy the place to buy these drugs because through formal channels they may ask for your sexual partners and ask you to inform them of the illness.” (Aminata, Buyer- FGD 2)*

The issue of trust in the informal market was also raised in relation to long family traditions of using this market. Several of our participants said the reason they used Roxy were social, they are used to the market and have been socialized to do so by family members:

*“I grew up in a family that consumes drugs from Roxy. I have always consumed drugs from Roxy because my family has always used them. Thus, for me it is normal to use these drugs. In recent years, people have begun saying that street drugs are harmful, yet I have always used them, and they have always been good to me.” (Abdul, Buyer- FGD 1)*

Edmond a participant in the same FGD raised the same point adding a class dimension. To him, it is those of high social economic status who can afford to buy from pharmacies, who advocate that the informal market for medicines is harmful:

*“I’ve been living in this neighborhood for more than 20 years. My family and I have always bought our medicine here (talking about Roxy). It is the people from rich residential suburbs who keep saying that these drugs are dangerous because they can afford to buy their drugs at the pharmacy.” (Edmond, Buyer- FGD 1)*

In addition to keeping up with family tradition, participants also had a perception that their life is in the hands of God, and they cannot do much to protect its longevity:

*“You know what-we will all die one day. God is the one who decides the moment, but everyone will have to face death. It does not matter how someone dies, it could be an accident, it could be by being bitten by a poisonous snake, it could be anything. Whether you are treated with Roxy’s medicines or not, we will all die one day. Are those who buy pharmacy drug immortal?” (Paul, Buyer- FGD 1)*

Serge was in agreement with Paul and goes on to say even if there could be stories of people dying from medicines bought from *Roxy*, that cannot stop him from using this informal market:

*“As Paul just said, life is a gift from God. God is the one who can take it back whenever he wants to. So even thought this lady who died was one of my family members, I think it would not have prevented me from using drugs from Roxy.” (Serge, Buyer- FGD 1)*

This extract also shows how religious beliefs and poverty can influence people’s decision-making. In this case, believing in God contributes to the acceptability of using the drugs from the informal market.

### Selling Medicines at the Informal Market as Employment

As a market, *Roxy* also thrives because it provides employment for many. The sellers see this as a form of employment, something that sustains their families. Sellers were very upfront in the interviews that the most attractive feature of the informal market for medicines is the financial aspect:

*“I do not make millions, but I am not to be pitied. Whoever sells drugs and reinvests the money in other businesses will get rich. This is not a business in which we need to put all eggs in one basket because we do not know what tomorrow holds for this nature of business.” (Fatou, Seller- IDI)*

Assia, while appreciating that the work has helped her to take care of her family and keep it going, she also stressed that the informal market for medicines is an unpredictable and not a very safe industry:

*“I cannot tell you that it's an easy and safe job. I have suffered to get to where I am today, but this job has always helped me to feed my family and pay for my children’s schooling. I plan to stop this activity in a few years to open a hair salon, it will be less stressful.” (Assia, Seller- IDI)*

Another seller, Roger, revealed the communal nature of the business. It is a business that allows for the strengthening of communal, social, and kinship ties. This connects to the point raised earlier that social reasons contribute to the thriving of Roxy. For the sellers and users, the enterprise is more than just a business for profit making. It offers the sellers an opportunity to help close friends and relatives in need on occasions they may not have money:

*“Yes of course my family is aware of my activity. They even come to take the drugs here at my stand. I lose money when they come by because they don’t pay for anything, but you cannot charge the family when into a business because they are the reason why you are doing this.” (Roger, Seller- IDI)*

The sellers expressed satisfaction about the presence of the informal market essentially because this market provides them with an income. In a country where unemployment rate is high, the informal market for medicines is a source of livelihood and this contributes as to why this market thrives.

## Discussion

Despite repeated attempts by authorities to put an end to *Roxy*, it is still thriving, and it does not seem like it will stop soon. In this section we will use the AAAQ framework, our findings and literature in this field to discuss reasons why informal markets for medicines are thriving.

As demonstrated with our findings, affordability ranks high on why this market is preferred. This market is mainly used by the poor^6,7^ and it enables low-income earners to get access to medicines terms of pricing.^23^ A study conducted in Ivory Coast also reported that informal market for medicines is perceived to be cheaper and convenient to users.^24^ Studies conducted in Bangladesh also revealed that the most commonly cited reason for visiting the informal drugstore is its affordability.^2^

In addition to the affordability of the informal market, another advantage is that prices are negotiable. The informal market also offers flexible terms of payment as noted in this and other studies.^2^ In pharmacies, prices cannot be negotiated over the counter. This flexibility makes the informal market more accessible especially to those of low social economic status.^9^ Our study also showed that sellers and users overtime develop mutual friendship and trust, and this becomes beneficial to both but especially to buyers as they end up having access to medicines even without cash up front. Out of pocket expenditure is one of the main reasons why health and medical products remains inaccessible to most of the poor.^25^

Sellers and buyers also develop a sense of loyalty and solidarity to one another overtime, and in this regard, buyers can choose to buy at the same place or refer their friends and families to the same vendor while sellers can also reduce prices and offer flexible payment options which may even extend to barter trading. This phenomenon of barter trading was reported in Tanzania where medicines were sold in exchange of food or electronics.^26^ These multiple payment options were also found to be existing in Bangladesh.^27^

Given this, it is important to note that the informal drug sellers therefore possess many social advantages over the formal market^2^ and these make the informal market for medicines more accessible. The relationship between sellers and buyers at the informal market for medicines are more personal and closer, findings from this study has demonstrated the importance of this relationship. This relationship increases the sellers’ credibility and strengthens their business position as well as duty to the community, creating socially binding ties as studies have shown.^2^

Another factor in favor of informal market for medicines is that they are conveniently located and closer to the populations they serve, we have seen this in this study. Another study from Cambodia reported the same findings.^28^ In addition, to the issue of proximity, the informal market is always open, even after hours when the formal drugstores are closed. The proximity and flexibility in operating times therefore makes the informal market more accessible. It is also a common practice that users when in need can even visit houses of sellers or vice versa even deep into the night and still get or offer a service.^28^ These are additional advantages the informal market carries over the formal market.

Besides being physically and financially accessible, *Roxy* offers a broad range of medical products at one stop. As one of our participants Lassana noted, *Roxy* offers both modern and traditional medicines, a variety of products that cannot be found in pharmacies as other studies have also shown.^9^ *Roxy* on its own has over 8000 sellers packed within a one-km radius, this increases chances for users to find any type of medicine they are looking for, be it modern or traditional.

Although medical products are available and accessible at *Roxy,* evidence from literature shows that these products are not of good quality,^29^ however, buyers still find the quality acceptable. Something that should concern policy makers and public health experts, especially in the context of COVID-19 and vaccines roll-out.

### Limitations and Strengths

Limitation in the current study involves the study design, data collection and selection of participants. The study was first designed as a project for a master’s thesis with a limited scope, we then had to collect additional data during a second phase. The second phase was disrupted by COVID-19 and this limited the amount of data we could collect during this second phase. Another possible weakness but also a strength is we used purposive sampling, this allowed us to target the informants and participants with the right information, however, this might introduce some biases or exclusion of other potential participants whose views might have enriched this study. The other limitation to this study related to the theoretical framework we used the AAAQ. The framework comes from a human right thinking and as we used it in this study presumes that the access of medicines even on the informal market is for health promoting activities. We, therefore, did not cover the dimensions where informal markets are used as hubs for recreational drugs. Additionally, the framework, did not afford us the opportunity to illuminate further on the sociocultural aspects that emerged in the findings. Another study specifically focusing on sociocultural factors and using a more expansive social theory may cover these dimensions.

### Implications

This study shows and demonstrates that there is a ready market for medical products on the informal market. This is very worrying considering that the bulky of substandard or falsified medical products end up in the Africa region.^1^ Markets like *Roxy* end up being the destination for most of these substandard medical and pharmaceutical products. The challenge is exacerbated by the fact that this is a very lucrative but illicit industry.^3^ In the context of COVID-19, it is not farfetched to assume that substandard and falsified vaccines may find their way into this ready market.^11,14,15^ This, therefore, calls for authorities to investigate the shortcomings not only of regulatory frameworks but also in the health systems.

## Conclusion

This study set out to find out the factors or reasons that is making a local informal market for medicines in Ivory Coast to flourish. We found out that the medicines at *Roxy* are perceived to be convenient and affordable. Additionally, there are many social and cultural reasons that make this informal market more accessible and acceptable to the local communities. Besides this fact this informal market paradoxically met most of the criteria of availability, acceptability and accessibility, the market itself is a source of livelihood for many young people. Pharmaceutical experts interviewed raised known dangers posed by these informal markets for medicines and local fatal cases including deaths were highlighted in these experiences recounted in this study. Despite these obvious dangers, sellers and buyers remained adamant that the pros of the informal market for medicines outweighs its cons. This study raises a fast-growing public health threat, which calls for a holistic and multi-pronged approach. The traditional solution of concentrating on regulatory frameworks and sanctioning the sellers may work but is proving not to be enough.
